# A near fatal complication following stenting of thoracic aortic aneurysm

**DOI:** 10.4103/1817-1737.74275

**Published:** 2011

**Authors:** Waseem M. Hajjar, Sami A. Al Nassar, Ahmed Iftikhar, Ahmed N. Alaqeed

**Affiliations:** *Thoracic Surgery Unit, King Khalid University Hospital, College of Medicine, King Saud University, Riyadh, Saudi Arabia*

**Keywords:** Bronchial obstruction, pulmonary vessel compression, thoracic aortic aneurysm

## Abstract

Tracheobronchial obstruction along with compression of pulmonary vessels is a rare complication after stenting of aortic aneurysm. We present this rare situation in a young patient who underwent stenting of traumatic thoracic aortic aneurysm and developed this near fatal complication and also the conservative management plan which we adopted to manage this case.

Although the thoracic aorta is closely related to the bronchus, pulmonary atelectasis due to an aortic aneurysm is uncommon.[1–3] We review the case report of a patient with complete compression of left main bronchus after endoluminal stenting of the descending thoracic aortic aneurysm along with significant compression of the right pulmonary artery. To our knowledge, the impact of stenting in cases where the aneurysm is compressing the left main bronchus along with significant compression of right pulmonary artery is a very rare phenomenon.

## Case Report

A 19-year-old male had a history of road traffic accident 18 months back with severe head injury and pelvic fracture. He remained in the referring hospital for three months. After discharge he remained reasonably well except for occasional episodes of anterior chest pain and dyspnea on moderate exertion.

Recently he was admitted again through the emergency department with complain of severe chest pain along with shortness of breath. CT chest with intravenous contrast showed large aneurysm of the descending thoracic aorta distal to the origin of left subclavian artery causing compression on the origin of the left main bronchus, and on the right and left pulmonary arteries with no evidence of dissecting or contrast leak; however, the patient referred to the vascular surgical unit in our institute for further management.

After admission he complained of mild shortness of breath. His vital signs and his routine blood investigation were within normal limits. His chest radiograph showed normal bilateral lung fields.

He underwent endoluminal thoracic aortic stenting under general anesthesia. Postoperatively the patient started to complain of severe dyspnea and was desaturating. His immediate post-procedure routine chest X-ray revealed complete collapse of the left lung [Figure 1a]. An urgent CT chest with intravenous contrast showed endovascular stent in place, but the aneurysmal sac was completely occluding the left main bronchus with complete collapse of the left lung but with adequate perfusion. The aneurysmal sac also had a mass compression on the adjacent right main pulmonary artery, which was compressed and stretched maximum at its proximal portion [Figure 1b].

**Figure 1a:**
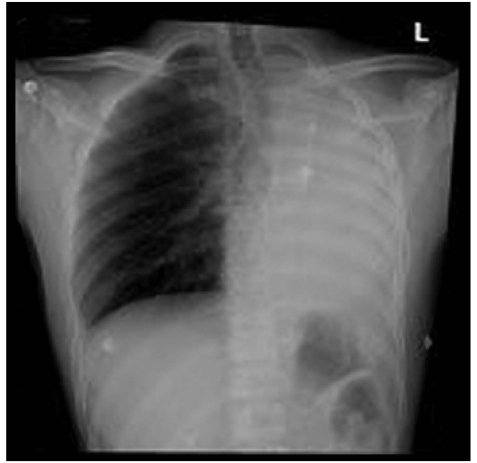
Complete collapse of left lung after endluminal stenting of aortic aneurysm

**Figure 1b:**
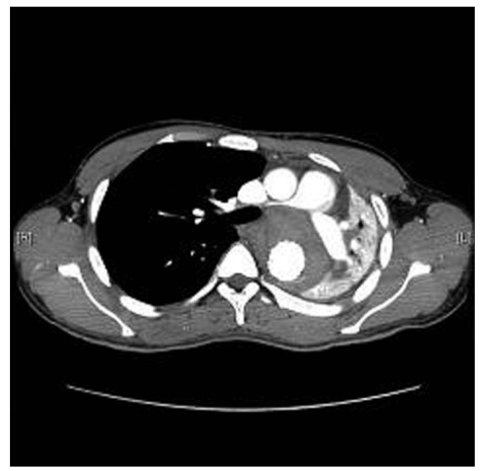
Complete collapse of left main bronchus by clotted aneurysm with significant compression of right pulmonary artery

Patient was stable hemodynamically and with relatively acceptable arterial blood gases.

He was monitored closely for possible emergency intervention with an endobronchial stent.

Although placement of an endobronchial stent in the left main bronchus can open the collapsed left lung, at the same time it may increase the pressure and can occlude the compressed right pulmonary artery supplying the only aerated right lung which could cause major morbidity, so, a conservative plan was adopted.

**Figure 2:**
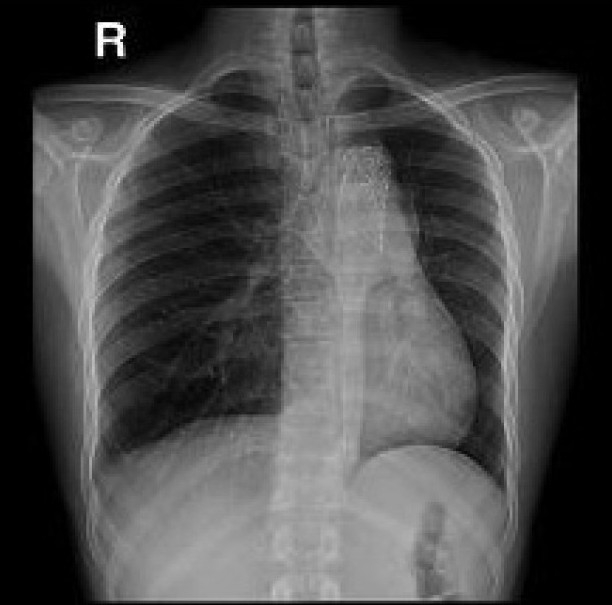
Fifteenth post-procedure day showing well-expanded left lung

After 18 hours the apex of the left lung started to open up radiologically which showed up on the chest X-ray. Patient’s arterial blood gas was improved gradually with the improvement of the expansion of the left lung over the next few days. He was discharged on the 15^th^ post-op day with complete expansion of the left lung [Figure 2]. He remained asymptomatic over the next fifteen months on his regular outpatient follow-up.

## Discussion

Traumatic aneurysms, occurring with increased frequency, are the result of sudden deceleration. Newer treatment approaches such as endovascular stent grafting appears to have lower perioperative morbidity and mortality rates compared to open surgery for the treatment of these aneurysms.[4]

In patients who have a history of blunt chest trauma, posttraumatic aortic aneurysms can occur and cause a left lung collapse by compressing on the left main bronchus.[5,6] This left lung collapse has been treated successfully by the implantation of an expandable stent to the left main bronchus.[7] However, if both, the thoracic aorta and the left bronchus are stented, the extrinsic pressure of the stented thoracic aneurysm may lead to a delayed aorto-bronchial fistula and pulmonary hemorrhage after stent implantation in the bronchus.[8]

In our case, the left main bronchus and both pulmonary arteries were compressed on the patient’s preoperative CT scan by the traumatic descending thoracic aortic aneurysm. Although endoluminal stenting by expandable metal stent was done successfully, this expanded metallic stent further increases the pressure through the aneurysmal sac (which is now hematoma). This leads to occlusion of left main bronchus completely and collapse of the left lung and also increases significantly the compression on the right pulmonary artery. Here we assessed the possible effects of stenting of left main bronchus on the right pulmonary artery that has already been compressed and stretched to its maximum at its proximal portion after the endoluminal stenting of the descending thoracic aneurysm. After detailed discussion we felt that this stenting of left main bronchus could lead to complete blockage of right pulmonary artery supplying the only aerated right lung, which could end up in a fatal outcome, and also there is possibility of formation of delayed aortobronchial fistula. As the patient was hemodynamically stable and was maintaining a relatively acceptable level of ABGs, a conservative approach was adopted with close monitoring. At the end the risk involved does not allow for placement of expandable metal stent in the left main bronchus in a relatively fit patient. We thought the most important aspect of this specific situation was the critical time of decision to hold the insertion of left bronchial implant and to give time for spontaneous improvement. The result was gradual opening of the bronchus with radiological improvement of the lung expansion.
